# A two-route CNN model for bank account classification with heterogeneous data

**DOI:** 10.1371/journal.pone.0220631

**Published:** 2019-08-19

**Authors:** Fang Lv, Junheng Huang, Wei Wang, Yuliang Wei, Yunxiao Sun, Bailing Wang

**Affiliations:** School of Computer Science and Technology, Harbin Institute of Technology at Weihai, Weihai, Shandong, China; Politechnika Krakowska im Tadeusza Kosciuszki, POLAND

## Abstract

Classifying bank accounts by using transaction data is encouraging in cracking down on illegal financial activities. However, few research simultaneously use heterogenous features, which are embedded in the time series data. In this paper, a two route convolution neural network TRHD-CNN model, fed with two types of heterogeneous feature matrices, is proposed for classifying the bank accounts. TRHD-CNN adopts divide and conquer strategy to extract characteristics from two types of data source independently. The strategy is proved able in mining complementary classification characteristics. We firstly transfer the original log data into a directed and dynamic transaction network. On the basis of that, two feature generation methods are devised for extracting information from local topological structure and time series transaction respectively. A DirectedWalk method is developed in this paper to learning the network vector of vertices used for embedding the neighbor relationship of bank account. The extensive experimental results, conducted on a real bank transaction dataset that contains illegal pyramid selling accounts, show the significant advantage of TRHD-CNN over the existing methods. TRHD-CNN can provide recall scores up to 5.15% higher than competing methods. In addition, the two-route architecture of TRHD-CNN is easy to extend to multi-route scenarios and other fields.

## Introduction

Almost any system that involves money and services can breed fraudulent activities, e.g. bank fraud, insurance fraud, telecommunication fraud, and e-commerce fraud. With economic development, the financial fraud activities seriously threaten the property security of customers. Identifying the anomaly bank accounts helps for providing clues to police in combating financial crimes. Therefore, the study of bank account classification has caused great interest among researchers in recent years [1]. In this paper, we call the research, classifying bank accounts, as CBA for short.

Due to its practical importance, classification algorithm has attracted extensive attention in many financial fraud scenarios. The existing classification methods can be categorized into three types: statistical based methods, time series based methods, and network based methods. Statistical based methods [2] utilize profile characteristics of the customers, made up by analyzing their trading behaviors, to generate the features used in classification. However, it has major limitation that relying on a specific fraud scenario, the methods are not able to detect unknown fraud patterns. The abnormal behavior, which changes over time, can be described by the time series based classification methods [3]. The two types of methods mine personal trading behavior characteristics from the historical data. Nevertheless, there are some activities that are normal individually but abnormal on group level. Therefore, the network based methods are proposed to solve these problems by use of the technique of the social network analysis [4]. Though the relationship data of the accounts is considered, the extracted network structure features, e.g. intimacy, modularity, are too coarse-grained to describe their relational structure in detail. In a word, the existing algorithms are mostly based on shallow features of the characteristics of accounts’ trading behavior.

**Fig 1:**
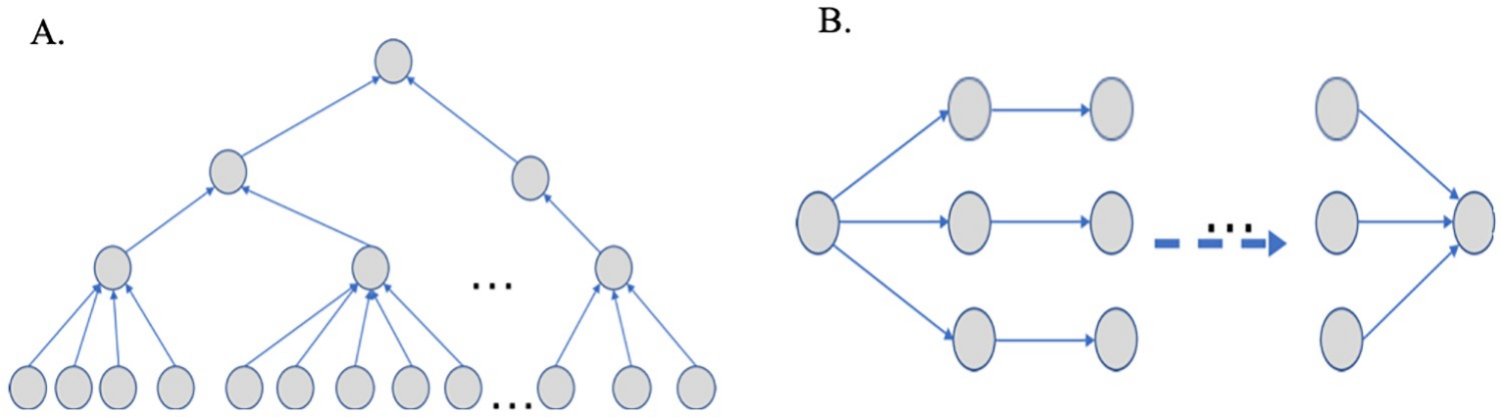
The examples of financial fraud activities accomplished by a group of members. (A) Trading behavior in MLM organization. (B) Trading behavior in money laundering organization. As shown in (A), the money transaction paths of MLM members are arranged in a tree-structured network. The transaction topological structure of members, especially members in the same layer, are similar to each other. A leaf node pays membership fee to its parent node and earns rebates from a upper member (not depicted). In addition, how many money members belonging to different levels earns decrease from top bottom. As depicted in (B), the money transaction paths are arranged in spindle-shaped structure. A large fund is firstly injected into the left node, and is split into a number of small funds. These small amounts are transferred in parallel and flow into the right node finally.

There are many kinds of financial fraud activities, e.g. money laundering, illegal multi-level marketing (MLM), accomplished by a group of people (*Examples* are depicted in Fig 1). In order to complete these activities, the members are controlled to trade in specifc orders and with certain counterparties. Therefore, from the perspective of a single person, its trading behavior is normal, however, in a broader perspective context, the trading behavior of the group is abnormal. The existing CBA methods cannot be used to detect this kind of the abnormal entities.

In recent years, deep learning [5] has drawn the researchers’ attentions due to its outstanding performance of feature learning. The convolution neural network (CNN) achieves impressive results in image classification [6], and credit card fraud detection [7]. The reasons why CNN architecture has been widely used can be listed as following: able to extract high-level semantic features automatically, and the structure is flexible to meet the needs of a wide range data types.

**Fig 2:**
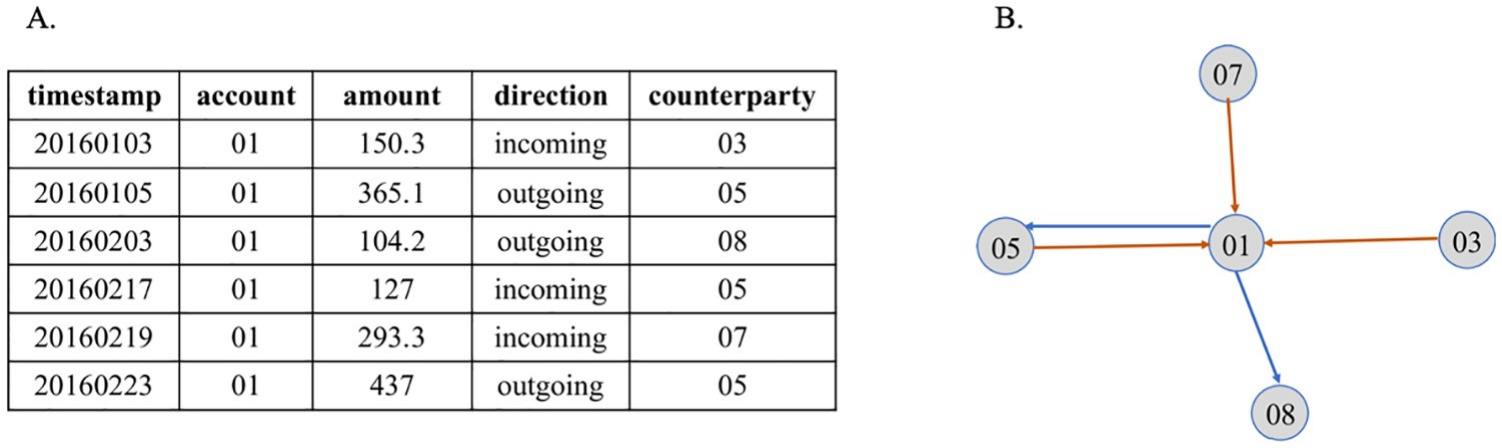
Transforming the original time series data into network relationship data. (A) Example of transaction log. (B) Relationship between the accounts.

In this paper, we proposed a two-route CNN classification model TRHD-CNN, fed with two types of heterogeneous data, to classify the bank accounts without using manual selected features. The framework of TRHD-CNN can be divided into two substructures orderly, i.e. *convolutional component and classification component*. The convolutional component contains two parallel convolution-pooling layer structures, which are employed to extract high-level features from two kinds of data separately. The outputs of the convolutional component are concatenated to train the classification model. The time series transaction data can be embedded into a weighted directed transaction network. As shown in Fig 2, the transaction data utilized in this paper is in form of the transaction log data. The front structure of TRHD-CNN extracts classification features from network structure and the account’s historical transaction data. In order to represent the network information, the method of network representation learning (NRL), DeepWalk, is proposed in [8] to encode network structure into a low-dimensional space. The NRL technique is proved to be successful in vertex classification. Therefore, a *network vector generation method* DirectedWalk, an improvement of DeepWalk [8], is devised to learn the latent relationships in the directed and dynamic network. The technique of NLP is utilized in obtaining the vertices representations. Thus that, for an account, the network vector matrix of its neighbor vertices is seen as the local topological feature, which is inputted into one route of the convolutional component.

Furthermore, the time series transaction features of an account is processed into a matrix, composed of the transaction amounts with its counterparties, and is fed into another route. Considering the minimum semantic units of the two feature matrices are quite different, we exploit different convolution-pooling layer structures to extract the most discriminative classification features. The TRHD-CNN model trains the two types of heterogeneous with a divide and conquer structure. Its classification component concatenates high-level features output from the convolutional component and optimizes the features together by using supervision information. The experimental results show that the TRHD-CNN model significantly outperforms the existing methods in terms of accuracy, recall and F1-score.

The main contribution of this paper can be listed as follows. (1) We build a weighted directed transaction network, which embeds the transaction relationship of bank accounts. (2) A network vector generation method DirectedWalk is proposed to extract the local topological relationship of bank accounts. (3) We build a two-route CNN model TRHD-CNN to classify bank accounts by using two kinds of heterogeneous data. TRHD-CNN can be easily expanded to other fields owing to its divide and conquer structure. (4) The experimental results show that TRHD-CNN outperforms the existing methods significantly.

The rest of this paper is organized as follows. Related work is reviewed in Sect. 1. In Sect. 2, we propose the generation methods of local typological feature matrix and time series feature matrix respectively. TRHD-CNN model is created in Sect. 3. The performance evaluation of TRHD-CNN is analyzed in Sect. 4. Sect. 5 concludes the paper, and finally Sect. 6 describes our future work.

## 1 Related work

The classification method is one of the most widely used methods in detecting financial fraud [9]. In this filed, traditional methods divide the classification task into two separate parts, i.e. feature mining and classifying. This section reviews the existing classification methods corresponding to financial fraud scenarios, and the multi-input CNN models.

### 1.1 Traditional classification method in financial fraud detection field

In recent years, different types of financial frauds and different fraud detection techniques are studied widely. The classification methods, according to the data they focus on, can be categories into three groups: statistical based methods, time series based methods, and network based methods.

The statistical based methods exploit the statistical features from the historical data for profiling an entity. In order to detect the credit card fraud, [10] devises a number of decision tree and SVM models, using the trading characteristics which are extracted from the continual monitoring data of an account’s trading records. The records contain geographical locations, transaction date, and Merchant Category Codes, etc. Any new incoming data is quantified and compared with the existing profiles to determine whether the difference is larger than a suspicion score. [11] mines frequent patterns from legal and fraud transaction data separately, and proposes a matching algorithm to find which pattern the incoming data is closer to. The Apriori algorithm [12], a classical algorithm in data mining, is utilized to return a set of frequent itemsets. Similarly, the technique of fuzzy association rule mining is exploited by [13] to extract knowledge useful for detecting credit card fraud accounts. Maes et al. [14] performs experiments, by using Bayesian belief networks and artificial neural networks, on a new transaction data to identify whether it is fraudulent or not. It is demonstrated that the Bayesian network has a better fraud detection capability.

The time series based methods use time series characteristics of transaction data to depict the trading pattern of an entity. [3] conceives a developed time series decomposition method EMD (Empirical Mode Decomposition) to extract the fluctuation features. The complex financial time series data is decomposed into some local detail parts and one global tendency part. [3] introduces the concept of peer group comparison, which is implemented by using a novel linear segment approximation method. [15] proposes a sequence matching method to detect the suspicious activities of an account according to its own transaction trend and temporal pattern. To create the temporal sequence of an account, [15] takes two kinds of information into consider, i.e. the historical transaction information and information from the account’s peer group. [16] considers the transaction data of a credit card as a data stream, and classifies the fraudulent users by using an expanded Very Fast Decision Tree (VFDT) method.

The network based methods, on the basis of a created transaction network, analyze the suspicious activities involves a cooperative group. Redmond et al [17] employ network analysis method in detecting the time-related suspicious groups in P2P (peer-to-peer) lending system. [17] creates a directed loan network with parallel edges, in which each edge corresponds to a timestamp. In the temporal network, the characteristics of variation in local structure are used to detect the suspicious active subgraphs, which occur during a short time interval. The experimental results demonstrate the existence of three dense structures, i.e. cliques [18], trusses [19] and FOFI. [20] describes a classification system that is capable of analyzing group behaviors by using network analysis method and supervised learning method together. A transaction network is modelled by [20], in which vertices are parties, edges mean relationships, and the communities are defined as near-k-top neighbors. Furthermore, an ego-centric approach is taken to build the communities in a bottom-up processing. Finally, the author creates a SVM and a random forest classifiers by using four kinds of community-related features, i.e. demographic, network, transaction and dynamic features. In order to identify illegal pyramid selling accounts, [21] builds a telecommunications network and creates the ego networks for different kinds of users. It proves that the ego networks of normal and service users are far different from that of the MLM members. The visualization of the ego network of a MLM member is shown as a tree-like structure. Based on the characteristics of MLM transactions, six network attributes are quantified and being used to detect the MLM members.

***Summary*** Most of the statistical features are extracted on the basis of analyzing the knowledge of the specific suspicious cases, [10] [13]. Table 1 summarizes the researches on methods and techniques in financial fraud detection field. The significant drawback is their weak scalable. Complemented with time series characteristics, the users’ trading pattern is expressed more sufficient in [15]. The challenge of time series based methods is how to design a sequence matching method, which is low in complexity but high in accuracy. The network analysis techniques are employed in detecting the suspicious accounts, owning abnormal collective behaviors [19] [20]. However, the employed network features are too coarse-grained to achieve a satisfying classification results. In order to capture the intrinsic patterns, [7] proposes a CNN-based detection framework to learn latent trading patterns from labeled data. In [7], a novel feature trading theory is devised and the one-dimensional transaction data is transformed into a feature matrix.

**Table 1:** Research on methods and techniques in financial fraud detection field.

Data type	Technique	Fraud area	Reference
Statistical features	Decision tree and SVM, Aprior algorithm, fuzzy association rule mining, bayesian belief network and artifical neural network	Credit card fraud accounts	[10], [11], [13], [14]
Time series features	EMD and peer group comparing, sequence matching, VFDT	Suspicious financial transaction, suspicious activites and accounts, credit card fraud accounts	[3], [15], [16]
Network features	Network analysis method, network analysis method and SVM+random forest, ego-network	P2P members, suspicious active subgraphs, MLM members	[17], [20], [21]

### 1.2 Multi-input CNN models

In order to extract more discriminative classification features from more perspectives, many CNN models with multi-input structures are proposed by researchers. [22] presents a handwritten digit recognition method based on cascaded heterogenous CNNs. Each CNN structure is built to recognize a proportion of input samples, and the following CNN is fed with the rejected samples. The reliability and complementation of heterogeneous CNNs are investigated in [22]. Simonyan et al. [23] extend deep Convolutional Networks (ConvNets), a state-of-the-art still image representation, to a two-stream ConvNet architecture for recognizing actions in video data. The two-stream ConvNet structure incorporates two separate ConvNet-style networks, training the spatial stream and the temporal stream together, to a multi-task learning framework. The complementray of the two recognition streams are demonstrated by the experiments results. [24] devises a multi-column deep neural network (MCNN) for crowd counting from an individual image by using three columns of CNNs. Three filters of different sizes are used in the three columns to adapt to the large variation in people size of an image.

***Summary*** An additional CNN structure is helpful for extracting complementary features for classifying samples. However, in [22] [23] [24], the types of convolution filters of multiple CNNs are the same. In this paper, two kinds of heterogenous data, i.e. network relationship data and time series data, are processed into two feature matrices and being used to describe the trading patterns of an accounts. Due to the fact that the adjacent elements’ relationships in those two matrices are quite different, each CNN inputs requires a distinct convolution filter. In this paper, we propose a TRHD-CNN model that contains two different CNN substructures to solve the aforesaid problem.

## 2 Preliminaries

In this section, the problem definitions are given first. Then, we devise a DirectedWalk algorithm for learning network vector of the accounts. Subsequently, the local topological feature and time series feature of accounts are formed into two matrices respectively.

### 2.1 Problem definition

This part gives the formal definitions of bank account classification.

**Definition 1** (***CBA problem***). *Given the bank account set A* = {*a*_1_, *a*_2_, …, *a*_*n*_} *and its category label set C* = {*c*_1_, *c*_2_, …, *c*_*m*_}, *where a*_*i*_ *denotes the ith bank account and c*_*j*_ *means label of the jth category*. *The CBA problem is to find the Cartesian product set D* ⊆ *A* × *C*, *where* ∀*i*, *j*, (*a*_*i*_, *c*_*j*_) ∈ *D*, 1 ≤ *i* ≤ *n*, 1 ≤ *j* ≤ *m*, *if and only if the category label of a*_*i*_ *is c*_*j*_.

Seen the accounts as vertices, the transaction relationships between accounts as directed edges and the transaction information as edge weight, the information of the transaction data can be created into a directed and dynamic network. We define the network as bellow.

**Definition 2** (***bank account transaction network***). *Given G* = (*V*, *E*, *W*, *T*), *where V* = {*v*_1_, *v*_2_, …, *v*_*n*_} *denotes the set of vertices*, *E* = {*e*_*ij*_}, *E* ⊆*V* × *V and* 1 ≤ *i* ≤ *n*, 1 ≤ *j* ≤ *n means the set of edges*. *The e*_*ij*_ = < *v*_*i*_, *v*_*j*_ > *represents the directed weighted edge from v*_*i*_ *to v*_*j*_, *if at least one transaction from v*_*i*_ *to v*_*j*_ *occurred*. *Then*, *we define W* = {***w***_*ij*_} *as the set of all of the weight information*, *and T* = {***t***_*ij*_} *means the set of all of the timestamp information*, *where* $w_{ij}=\left(r_{ij}^{\left(1\right)},r_{ij}^{\left(2\right)},...,r_{ij}^{\left(Nt\right)}\right)$, *and* $t_{ij}=\left(t_{ij}^{\left(1\right)},t_{ij}^{\left(2\right)},...,t_{ij}^{\left(Nt\right)}\right)$, ***w***_*ij*_ ∈ (*R*^+^)^*Nt*^ *is the weight vector of e*_*ij*_, *R*^+^ *is the set of positive real numbers*, *Nt is the total number of transactions*, $r_{ij}^{\left(k\right)},k=1,2,...,Nt$ *means the kth transaction amount from v*_*i*_ *to* *v*_*j*_, *and* $t_{ij}^{\left(k\right)}\in t_{ij}$ *is the timestamp of* $r_{ij}^{\left(k\right)}$.

**Fig 3:**
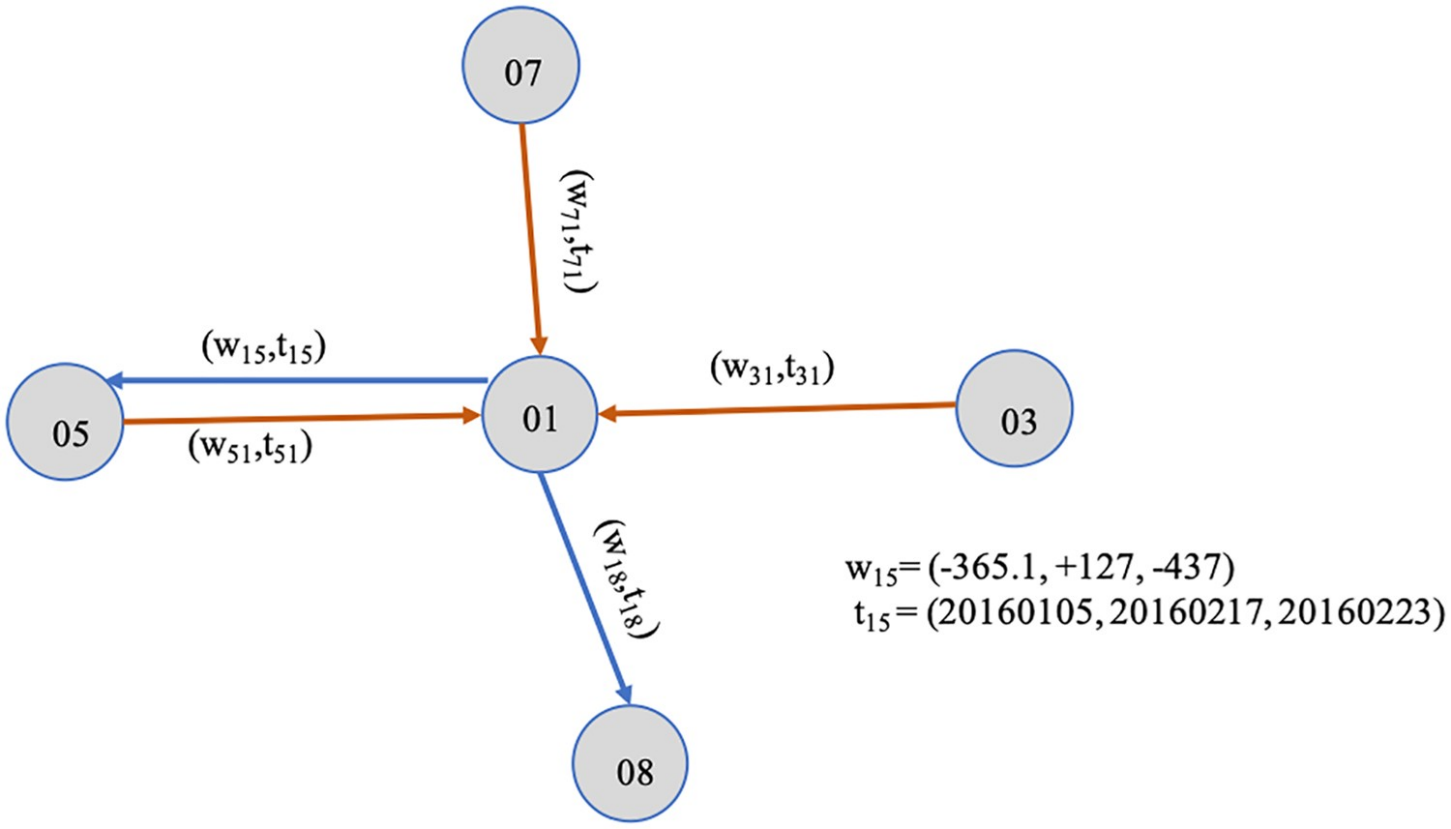
An instance of Definition 2.

According to the Definition 2, the network built by the information given in Fig 2 can be depicted in Fig 3. Given *V* = 01, 03, 05, 07, 08, *E* = *e*_15_, *e*_18_, *e*_31_, *e*_51_, *e*_71_, the weight and timestamp information are embedded in the transaction network. For instance, the information of *e*_15_ is denoted as a 2-tuple (*w*_15_, *t*_15_), where *w*_15_ = (−365.1, + 127, −437) and *t*_15_ = (20160105, 20160217, 20160223). For *w*_15_, the *k* equals 1, 2, 3, and the sign of each $w_{ij}^{\left(k\right)}$ mean the direction of money flow, the minus sign of $w_{ij}^{\left(1\right)}$ means *i* delivers money to *j* and a plus means *i* gains money from its counterparty. Thus, all of the transaction records are translated into the directed and dynamic network, e.g. the last record in Fig 2 is expressed as $\left(w_{15}^{\left(3\right)},t_{15}^{\left(3\right)}\right)$.

**Definition 3** (***neighbor vertex set***). *Given G*, *and neighbor radius h*, *for* ∀*v* ∈ *V*, $N_{v+}^{\left(h\right)}=\left\{w|<w,v>\in E\right\}$ *represents its h step neighbor vertices exist incoming transactions*, *and* $N_{v-}^{\left(h\right)}=\left\{u|<v,u>\in E\right\}$ *means the reverse vertex set*. *Therefore*, *the neighbor vertex set of v is denoted as* $N_{v}^{\left(h\right)}=N_{v+}^{\left(h\right)}\cup N_{v-}^{\left(h\right)}$.

**Fig 4:**
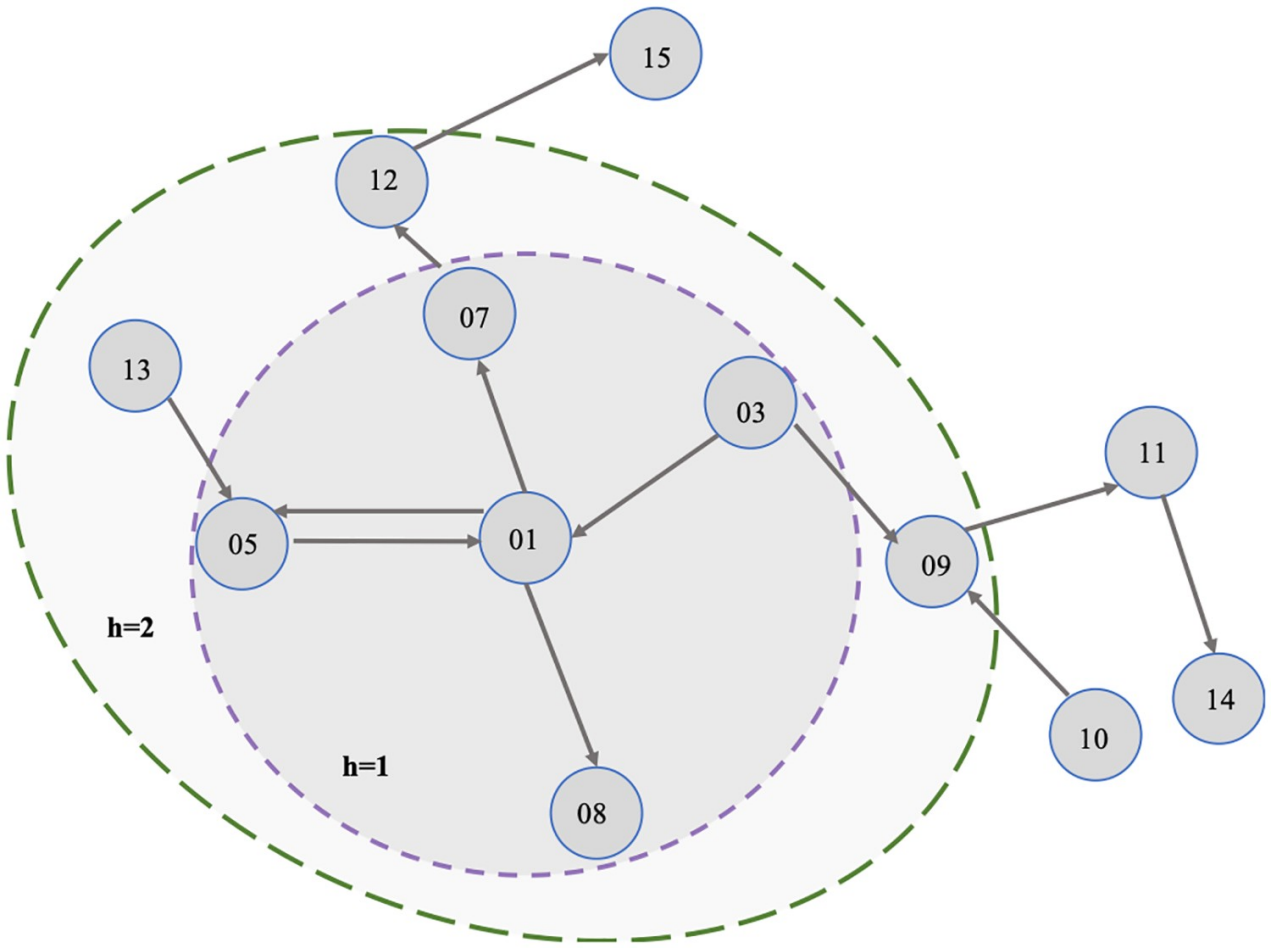
An instance of Definition 3.

According to the Definition 3, given the local typological structure of vertex 01, showing in Fig 4, the $N_{01}^{\left(1\right)}$ is the set of vertices in the inside circle and $N_{01}^{\left(2\right)}$ contains the vertices in the outside ellipse. As illustrated in Fig 4, $N_{01}^{\left(2\right)}=01,03,05,07,08,09,12,13$, in which $01,03,09,05,13\in N_{v+}^{\left(2\right)}$ and $01,05,13,07,12,08\in N_{v-}^{\left(2\right)}$.

### 2.2 Generating the local topological feature matrix

Economic crime investigators believe that it is helpful to consider the vertex itself and its neighbors comprehensively in determining whether a vertex is fraudulent. In other words, for any *v* in *G*, which category it belongs to is closely related to its local topological structure *G*_*v*_.

We propose a DirectedWalk algorithm, an improvement version of DeepWalk [8] in directed and dynamic network, to learn the network vectors of accounts. Based on this, a method to generate an account’s local topological feature matrix is presented by constructing its transactional relationships.

#### 2.2.1 DeepWalk

To capture the network topology information, [8] proposes a DeepWalk approach, which learns features that describe the graph structure. The optimization techniques, originally designed for language modeling, are used for learning social representations of a graph’s vertices. DeepWalk takes an graph as input and produces its latent structure representation as an output. The learned structural features are used in many applications such as network classification and anomaly detection with outperform results.

DeepWalk algorithm, borrowing the concept in language modeling, considers a set of short truncated random walks as its own corpus, and the graph vertices as its own vocabulary. There are two main components, a random walk generator and an update procedure. Given graph *G* as input, the random walk generator samples uniformly a random vertex *v* as the root of the random walk *W*_*v*_. For each vertex *v*, being the start of *γ* random walks, |*W*_*v*_| vertices are selected in order randomly. Aiming at updating the vector representation of *v*, Skip-Gram [25] is utilzed to maximize the probability of its neighbors in the walk *W*_*v*_. Inspired by DeepWalk, we propose a DirectedWalk algorithm to learn network vectors of accounts in the transaction network.

#### 2.2.2 Learning the network vector

We generate a corpus and a vocabulary from the directed and dynamic network, which is the only required input for learning the network vectors. Give *G* = (*V*, *E*, *W*, *T*), the vertex set *V* is considered as its own vocabulary, and the directed sequential transaction paths are seen as its own corpus. It is well known that, in DeepWalk, the relationship strength of two vertices is determined by the frequency they occur in an adjacent position in random walks. Here, as defined in Definition 2, the strength of the relationship between any two vertices *v*_*i*_ and *v*_*j*_ is determined by weight **w**_*ij*_ of *e*_*ij*_ and **w**_*ji*_ of *e*_*ji*_. Moreover, in *G*, the order of vertices that passed by a transaction path are not random but time-related. Therefore, we propose a DirectedWalk algorithm for learning the network vector of the transaction vertices. Each vertex can be seen as the start vertex of a directed walk with a maximum length of *l*. For the last visited vertex *v*, the walk will pass over all of its directed neighbors, only if *v* has trading with them within a time interval *τ*. The walk grows up iteratively until not satisfies the constraints.

**Algorithm 1** DirectedWalk(*G*, *l*, *τ*, *w*, *d*)

**Require**:

 network *G* = (*V*, *E*, *W*, *T*)

 maximum walk length *l*, timestamp interval *τ*

 window size *w*, embedding size *d*

**Ensure**:

 matrix of vertex rpresentations Φ ∈ *R*^|*V*|×*d*^

1: the directed corpus *NC* is initialized to be empty;

2: **for** *v*_*i*_ ∈ *V* **do**

3:  *p*_*i*_ = [*v*_*i*_], *p*_*i*_ ⊆ *P*_*i*_

4:  **while** ∃*p*_*i*_.*state* = *True* **do**

5:   NodeSentence *p* = *P*_*i*_.pop() (pop a *p* in its active state)

6:   *v* = *p*.*lastvertex*, *t* = *v*.*timestamp*

7:   retrieve *v*’s directed neighbor vertices ((*v*_1_, *t*_1_), …, (*v*_*m*_, *t*_*m*_)) from *G*

8:   **for** *j* ∈ *m* **do**

9:    **if** (∃*t*_*k*_ ∈ *t*_*j*_, *t* < *t*_*k*_ < *t* + *τ*) or (*len*(*t*_*j*_) = 1 and $t_{j}^{\left(0\right)}>t$) **then**

10:     *p* adds a new vertex (*v*_*j*_, *t*_*j*_)

11:     **if** *v*_*j*_ has no directed neighbor vertex or *len*(*p*) = *l* **then**

12:      *p*.*state* = False

13:     **end if**

14:     *P*_*i*_ = *P*_*i*_.*push*(*p*) (push *p* into *P*_*i*_)

15:    **end if**

16:   **end for**

17:  **end while**

18: **end for**

19: *NC* = *NC* ⋃ *P*_*i*_

20: **for** *c* ∈ *NC* **do**

21:  *SkipGram*(Φ, *c*, *w*)

22: **end for**

Line 2-18 in Algorithm 1 shows the core of our algorithm. The outer loop specifies the paths that start with each vertex and generates a time series ordering of the directed neighbor vertices. The state factor of *p*_*i*_ is initialized to *True* and will be reset to *False*, in condition that its last vertex has no directed neighbor vertex or the length attains *l*. On condition that all of the paths reach their *False* states, the generation procedure of *P*_*i*_ starting at *v*_*i*_ is completed. As depicted in line 8-16, for the last passed vertex *v*, the timestamp information is used to determine whether a vertex will be appended to *v*. In a summary, fixing a start vertex, the longer the weight vectors of the passed directed edges are the more walks will be produced by our DirectedWalk approach. Therefore, the frequent traders are more likely to exist in walks and appear in a window with high probability.

It is shown in line 21, the Skip-Gram model [25] is employed to maximize the co-occurrence probability Φ among the vertices in the time ordered walks. Skip-Gram, using the independence assumption, iterates over all possible collocations in directed corpus *NC* within the window *w*.

We finally obtain the optimal network vector representations of the vertices by using the same learning process of DeepWalk. DirectedWalk maps the directed and dynamic transaction network into a *d*-dimensional vector space. The vertices that contain similar local directed topological structures are mapped into adjacent vectors.

#### 2.2.3 Constructing the local topological feature matrix

For ∀*v* ∈ *V*, given $N_{v}^{\left(h\right)}$ in *G*, the local topological structure of vertex *v* is the subgraph *G*′ of vertex set $N_{v}^{\left(h\right)}\cup \left\{v\right\}$. In Sect. 2.2.2, the structure of *v* in *G*′ is embeded into a *d*-dimensional network vector **v**^*t*^. Having calculated the Euclidean distance between **v**^*t*^ and $v_{i}^{t}\in N_{v}^{\left(h\right)}$, we store the top *k* nearest $v_{i}^{t}$ in ascending order. This yields the local topological feature matrix *T*_*v*_ as follows,

$$T_{v}={\left(v_{0}^{t},v_{1}^{t},v_{2}^{t},...,v_{k}^{t}\right)}^{T},T_{v}\in R^{(k+1)\times d}$$ (1)

where, $v_{i}^{t}$ (1 ≤ *i* ≤ *k*) is the network vector of $v_{i}\in N_{v}^{\left(h\right)}$, while $v_{0}^{t}$ denotes the network vector of *v* itself.

### 2.3 Creating the time series feature matrix

For each account, the time series records are consist of incoming and outgoing direction transactions, and each record is composed of three kinds of information, i.e. two accounts, a timestamp and transaction amount. In a real life scenario, the latent information in its transaction sequence is helpful for determining which category an account belongs to.

According to the Definition 2, given *u*, *v* ∈ *V*, the weight of **e**_*uv*_ and **e**_*vu*_ are denoted as **w**_*uv*_ and **w**_*vu*_ repectively. For instance, **w**_*uv*_ contains the transaction amounts from *u* to *v* in chronological order. Therefore, all the incoming transactions of *v* are represented as set *S*_−*v*_ = (*w*_1*v*_, *w*_2*v*_, …, *w*_*nv*_) ($n=\Sigma_{i\in N_{v}^{\left(in\right)}}|w_{iv}|$), and its outgoing transactions are denoted as set *S*_*v*−_ = (−*w*_*v*1_, −*w*_*v*2_, …, −*w*_*vm*_) ($m=\Sigma_{i\in N_{v}^{\left(out\right)}}|w_{vi}|$). Integrating the above two sets and sorting its elements by their occurrence timestamps, the time series vector of *v*, in length of *N* = *n* + *m*, is expressed as:

$$v^{s}=\left(w_{1},w_{2},\ldots ,w_{N}\right),w_{i}\in \left\{S_{-v}\cup S_{v-}\right\},1\leq i\leq N.$$ (2)

In this paper, the time series feature of vertex *v* is created by its top *k* nearest neighbor vertices, which are obtained from Sect. 2.2.2. The feature matrix *W*_*v*_ is described as follows,

$$W_{v}={\left(v_{0}^{s},v_{1}^{s},v_{2}^{s},\ldots ,v_{k}^{s}\right)}^{T},W_{v}\in R^{\left(k+1\right)\times d_{w}}$$ (3)

where, $d_{w}=\max_{i\in [0,|V|]}\left(\right|v_{i}^{s}\left|)\right)$ denotes the maximum length of $v_{i}^{s}$, which belongs to *V* ∈ *G*. And then, we pad the empty positions in matrix *W*_*v*_ with 0.

Thus far, the local topological and time series information of bank accounts are embedded into feature matrices respectively. These two features are two types of heterogenous data in the same matrix format, which can be used as the input of a CNN model.

## 3 The CNN classification model

In our earlier work, a bank account classification model MHD-CNN has been presented, which splices the two matrices up and down into a concatenated matrix, and then classifies the new matrix by using a CNN structure. In order to achieve better classification performance in lower computational complexity, a novel two-route CNN classification model TRHD-CNN is devised in this paper. This section begins with a review of the framework of MHD-CNN, summarizing the defects in previous study. Then, the TRHD-CNN model is described in detail.

### 3.1 The MHD-CNN model

**Fig 5:**
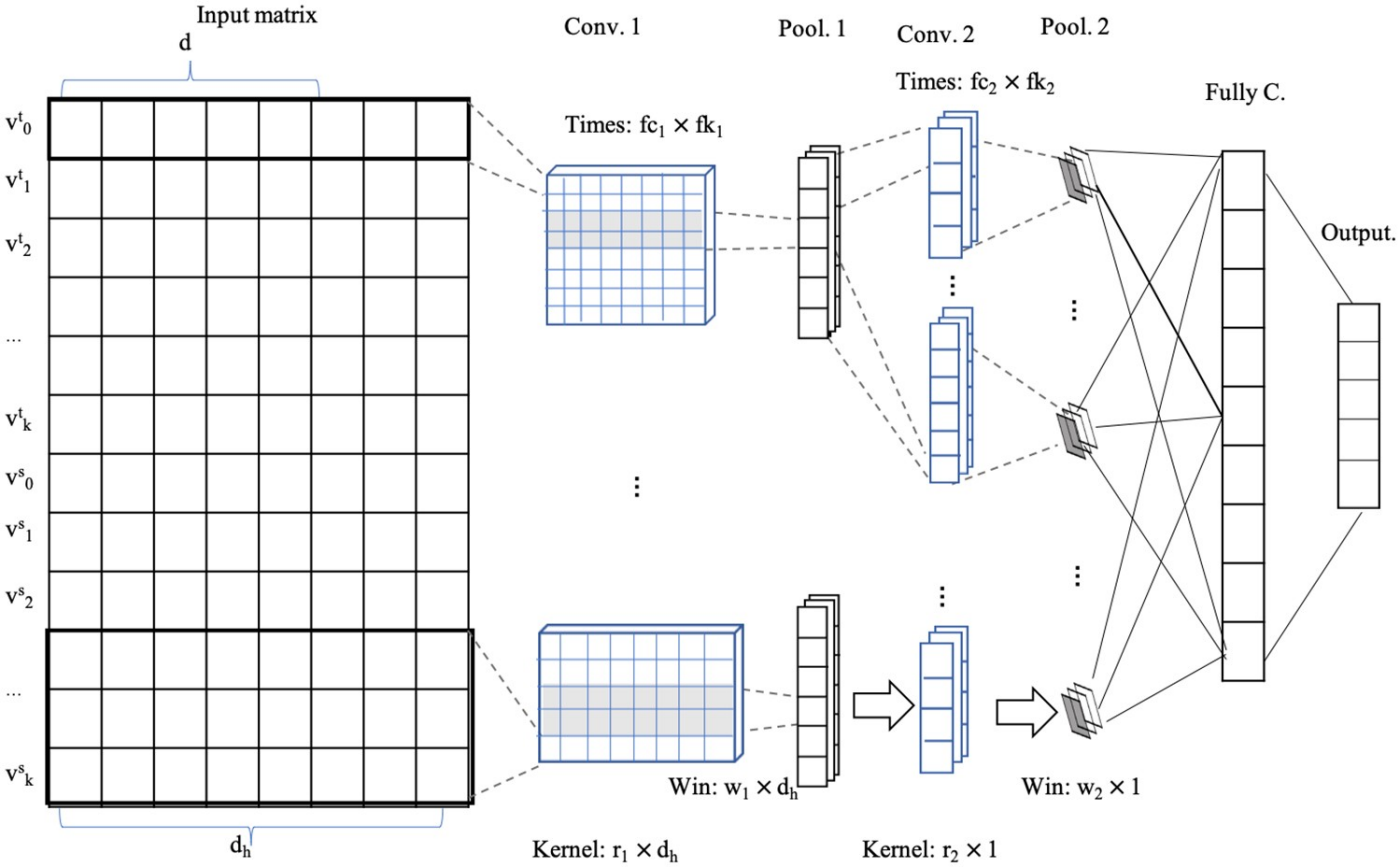
The WHD-CNN classification model using concatenated heterogeneous data. There are two convolutional layers and each is followed by a pooling layer. *Conv*.1 denotes the first convolutional layer, which contains *fc*_1_ kinds of convolution kernels. Each kernel, in size of *r*_1_ × *d*_*h*_, generates *fk*_1_ feature maps. *Pool*.1 represents the first pooling layer, which reduced the shape of a feature map but does not change the number of feature maps. The window size, in *Pool*.1, is *w*_1_ × *d*_*h*_. In *Conv*.2, each kind of input feature map is calculated by *fc*_2_ kinds of convolution kernels, in which each contains *fk*_2_ convolution kernel. *FullyC*. means the fully connected layer and *Output*. is the output layer.

For ∀*v* ∈ *V*, the concatenated matrix *H*_*v*_ is described as in formula 4,

$$H_{v}={\left[\begin{array}{c} T_{v}\\ W_{v} \end{array}\right]}^{T},H_{v}\in R^{2\left(k+1\right)\times d_{h}}$$ (4)

where, *d*_*h*_ = max{*d*, *d*_*w*_} is the maximum value of column numbers of *T*_*v*_ and *W*_*v*_. The empty positions of matrix *H*_*v*_ is padded with 0. Obviously, *H*_*v*_ expresses the characteristics of vertex *v* with two kinds of heterogeneous data comprehensively. Taking *H*_*v*_ as input, the framework of MHD-CNN is depicted in Fig 5.

Considering that each row of *T*_*v*_ in matrix *H*_*v*_, defined in formula 1, means a network vector of a certain neighbor vertex, one row of *H*_*v*_ should be taken as the basic unit when constructing the convolution kernels. That means the convolution kernel of MHD-CNN should cover at least a row of *H*_*v*_. Similar to the *n*-gram technique in NLP, our convolution kernels are designed in *n*-vertex format, e.g., one-vertex, two-vertex and three-vertex. In this way, the local shallow features relating to a different number of vertices are extracted into high level features. As depicted in Fig 5, the used parameters of the convolutional layers are set as follows:

- The number of types of convolution kernels: *fc*_1_ and *fc*_2_ are set as 3.
- The stride length for each convolution kernel is set as 1.
- The number of each type of convolution kernel: *fk*_1_ and *fk*_2_ are is set as 30.
- The size of each convolution kernel: *r*_1_ and *r*_2_ are the same and range from {1, 2, 3}.

Although heterogeneous data are used to increase the discriminative classification features, MHD-CNN also has some problems, which are listed as bellow.

- MHD-CNN exploits same types of convolution kernels on two kinds of heterogeneous data, and it cannot fully mining their unique characteristics.
- The integrated input matrix doubled the size of model parameters, resulting in high computational complexity.

Therefore, we propose an improved version of MHD-CNN, which possesses a parallel convolution structure to extract two kinds of characteristics independently.

### 3.2 The TRHD-CNN model

**Fig 6:**
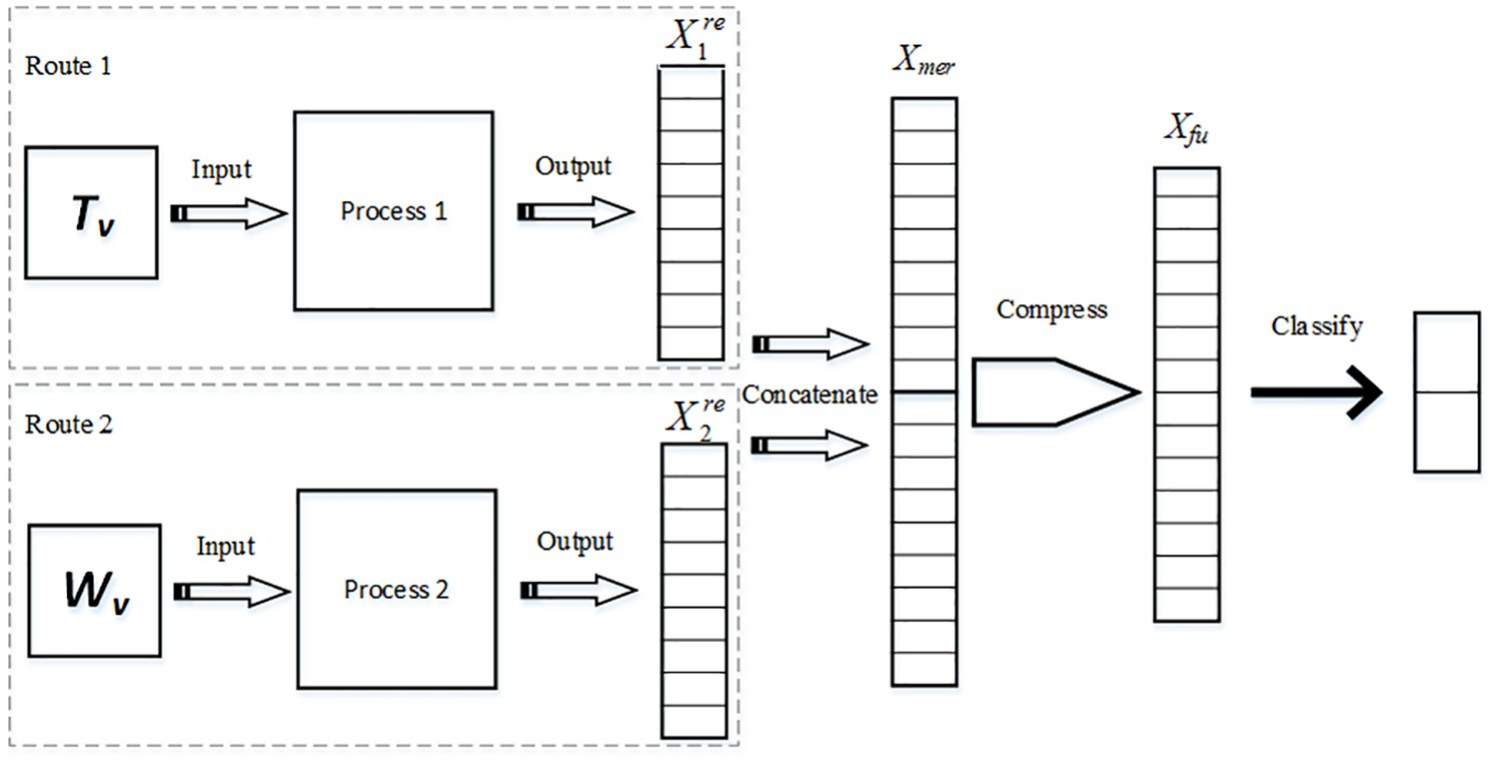
The architecture of the TRHD-CNN model. Given an account *v*, the feature matrices *T*_*v*_ and *W*_*v*_ are input to the model through different routes. Features in *T*_*v*_ are mapped into vector $X_{1}^{re}$ by Process 1 and those in *W*_*v*_ are mapped into $X_{2}^{re}$ through Process 2 similarly. Being concatenated into *X*_*mer*_, the features from $X_{1}^{re}$ and $X_{2}^{re}$ are compressed into a shorter vector *X*_*fu*_ by a fully connected layer. The output layer can be seen as a classifier that maps the features of *X*_*fu*_ into two category labels.

The novel two-route CNN classification model TRHD-CNN employs different convolutional and pooling mechanisms on two kinds of data. Its architecture is depicted in Fig 6.

#### 3.2.1 The first route in the TRHD-CNN architecture

The first route (i.e., Route 1 in Fig 6) extracts the local topological structure features of an involving account from its input matrix *T*_*v*_. As described in Sect. 2.2.3, the row vector of *T*_*v*_, is the minimum unit in describing its local topological structure. Thus, the convolution structure of this route is similar to that of the MHD-CNN model, with the following process steps.

The first route of the TRHD-CNN model fed with *T*_*v*_ employs the same architecture as the MHD-CNN model, except from containing the softmax output layer (seen in Fig 5). Then, vector $X_{1}^{re}$, part of the output of the convolutional component of TRHD-CNN, will be used in the subsequent classification procedure. The structure of Process 1 is expalined as following:

1. Input layer. For ∀*v* ∈ *V*, the input data is the matrix *T*_*v*_.
2. Convolutional layer. The input matrix *T*_*v*_ is used as the feature map in the first layer. The convolution kernels are same with those used in MHD-CNN. For the *l*th convolutional layer, the processing function on each feature map is shown in formula 5,
  $$X_{i}^{l}=f\left(X^{l-1}*K_{i}^{l}+b_{i}^{l}\right)$$ (5)
  where, *f*(•) represents an activation function, the operator “*” expresses the convolution operation, *X*^*l*−1^ denotes an input feature map, $K_{i}^{l},\left(1\leq i\leq 3\right)$ denotes the *i*th convolution kernel as mentioned in Sect. 3.1, and $b_{i}^{l}$, means the bias vector. Therefore, the kind of output feature maps is ∑_*i*_ *K*_*i*_ times that of the input.
3. Pooling layer. The processing, on the feature map of the *l*th pooling layer, is shown in formula 6,
  $$X_{i}^{l}=X_{i}^{l-1}\cdot P_{i}^{l}+b_{i}^{l}$$ (6)
  where, operator “⋅” represents a pooling function, which is used for downsizing the *i*th input matrix $X_{i}^{l-1}$. The notations $P_{i}^{l}$ and $b_{i}^{l}$ denote the weight matrix and bias vector respectively. The number of feature maps is not changed by the pooling procedures.
4. Connection layer. In this layer, the input feature maps, each in size of 1 × 1 and is obtained from the last pooling layer, are concatenated into a one-dimensional vector $X_{1}^{cc}$.
5. Fully connected layer. This layer compressed the vector $X_{1}^{cc}$ into a shorter vector **X**^*re*^ in length of *K*_1_ by using a fully connected structure.

#### 3.2.2 The second route in the TRHD-CNN architecture

**Fig 7:**
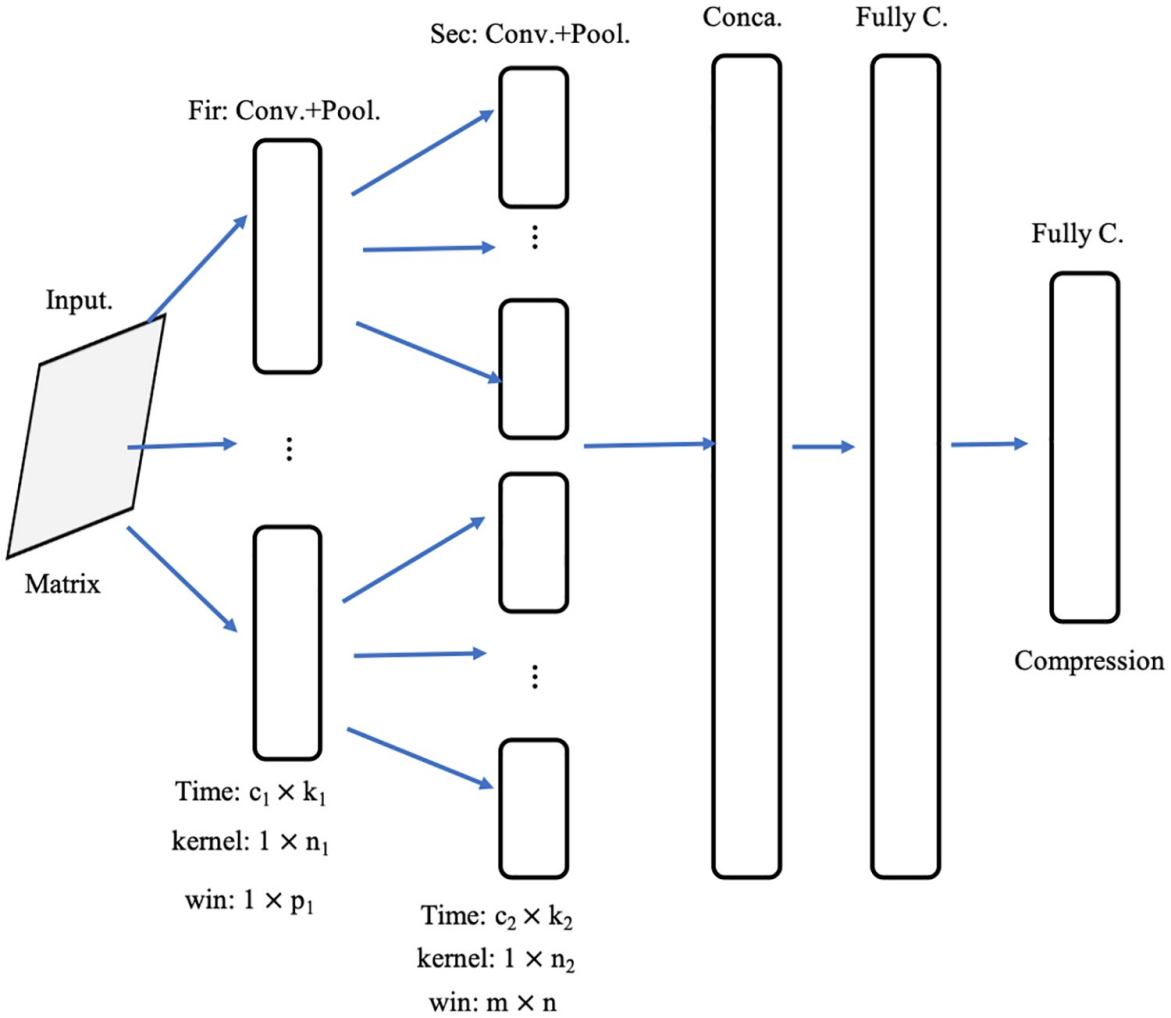
The second route structure in the TRHD-CNN model. *Input*. is the input layer. *Fir*: *Conv*. + *Pool*. is the first convolutional layer and its following pooling layer, which contains *c*_1_ kinds of convolution kernels and generates *c*_1_ × *k*_1_ output feature maps. The window size of this pooling layer, *win*, is 1 × *p*_1_. In the *Sec*: *Conv*. + *Pool*. part, each kind of input feature map, corresponding to a convolution kernel of *c*_1_, generates *c*_2_ × *k*_2_ output feature maps by using *c*_2_ kinds of convolution kernels. And, the pooling window, in size of *m* × *n*, down-samples each input feature map into an element. *Cona*. denotes the concatenation layer that splice the feature maps into a column vector. *FullyC*. represents the fully connected layer. The last *FullyC*. is utilized to compress the output into a lower dimensional vector.

The second route (i.e., Route 2 in Fig 6) is primarily used to extract discriminative classification features from the time series feature matrix of a vertex *v*, denoting as *W*_*v*_. The difference between *W*_*v*_ and *T*_*v*_ is that the minimum feature representation unit is a row vector for matrix *T*_*v*_, while for matrix *W*_*v*_ each data element does. Therefore, a new kind of convolution structure, different from the first route, is needed for extracting more useful information. The second route of TRHD-CNN is designed as following, shown in Fig 7.

1. Input layer. For any vertex *v*, the input data is its time series feature matrix *W*_*v*_. The length of the row vector of *W*_*v*_ is set as the longest transaction sequence in network *G*. *W*_*v*_ is a sparse matrix, for the reason that the trading frequencies of accouts are quit different.
2. Convolutional layer and pooling layer. As shown in Fig 7, the structure, two convolutional layers with each followed by a pooling layer, is built referring to the following details. Moreover, the trading behaviors of different accounts are independent, which are hidden in their time series transaction amounts. Therefore, the convolution kernels of the first convolutional layer, *c*_1_, is created in form of 1 × *n*_1_, *n*_1_ ∈ *N*_1_. Taking the matrix *W*_*v*_ as input, this first layer generates |*N*_1_| × *k*_1_ feature maps by using *k*_1_ convolution kernels on each input feature map. In order to extract the trading characteristics of a single vertex, the pooling window is created in 1 × *p*_1_ format. That means a pooling window does not slide over adjacent vertices. Similar to the first one, the second convolutional layer is also built in the multi-kernel structure. *c*_2_ kinds of convolution kernels are utilized, in which each in 1 × *n*_2_, *n*_2_ ∈ *N*_2_ format. Aim to describe the local region features of a feature map, we employ pooling windows in shape of *m* × *n* on the second pooling layer. Here, *m* and *n* equal the row and column values of the corresponding input feature map respectively. The processing of the convolutional layer and the pooling layer correspond to formula 5 and formula 6 in Sec. 3.1, respectively.
3. Fully connected layer. There are two fully connected layers in this route. The first one connects the |*N*_1_| × *k*_1_ × |*N*_2_| × *k*_2_ feature maps, generated by *Sec*: *Conv*. + *Pool*., into a one-dimensional vector $X_{2}^{cc}$. Subsequently, another fully connected layer is constructed to compress vector $X_{2}^{cc}$ and balance the effects of each route. $X_{2}^{cc}$ is reduced into $X_{2}^{re}$ of length *K*_2_, and then is used as a part of the input for the succeeding classification process.

#### 3.2.3 Concatenating the two routes of TRHD-CNN

This part describes the classification component of TRHD-CNN, including three portions: concatenation portion, compression portion and classification portion.

1. The concatenation portion. The vectors $X_{1}^{re}$ and $X_{2}^{re}$, come from the first route and the second route, are concatenated into vecto $X_{mer}=\left(X_{1}^{re},X_{2}^{re}\right)$.
2. The compression portion. A fully connected layer is used to compress **X**_*mer*_ into $X_{f}^{re}$.
3. The classification portion. The portion acts as the output layer of TRHD-CNN, outputs the category of a vertices *v* with $X_{f}^{re}$.

## 4 Experiments

### 4.1 Environmental settings

A deep learning server with GPU NVIDA LESLA P100 and 128G memory is adopted to improve the speed of training the CNN models. TensorFlow is selected to train the MHD-CNN and TRHD-CNN models.

### 4.2 Data set

In recent years, we have been exploring computational models to classify bank accounts in combating illegal pyramid selling. The department of economic investigation provides us with plenty of transaction data of real bank accounts. An instance of transaction records is shown in Fig 2. An account contains a lot of transaction records, each of which includes bilateral transaction accounts, timestamp, amount of money and transaction direction, etc. We sample out the transaction records belonging to 10145 bank accounts to form out dataset for training our model. There are 9270 normal accounts and 875 accounts involving a MLM organization respectively. As shown in Table 2, the number of transaction records generated by the normal accounts run up to 6732730 and the fraud records created by MLM members amount to 275804 rows. These MLM members are manually annotated as “illegal” by economic investigators. Before training the models, we filtered out some noisy data, i.e. deleting the duplicate records, incomplete records and the records whose transaction amounts no more than 50. Therefore, 1371914 records is filtered out from the set of normal accounts’ transaction records and 91341 records created by illegal accounts are deleted. In general, more than 5 million transaction records are used after denoising. In our experiment, all the accounts are mixed and five-fold cross validation strategy is adopted to evaluate the classification effective of our TRHD-CNN model. That means the dataset is divided into 5 portions and each time the model is trained with four portions as training set and the remaining portion as testing set. In other words, the proportion of training accounts and testing accounts is set as 4:1 in the training process.

**Table 2:** Characteristics of the dataset.

Dataset	Normal account	Fraud account
No. of accounts	9270	875
No. of edges	188359	39744
No. of transactions	6732730	275804

To obtain the optimal model, the back-propagation method [26] is used to update the hyper parameters. Aim to speed up the convergence rate of TRHD-CNN, we adopt the Mini-batch Gradient Descent (MBGD) method [27] in the iteration process. That means, the training process will stop under the condition that the objective function is stable at its minimal change. Note that, the hyperparameter of two routes are updated by joint training.

### 4.3 Experimental settings

For our TRHD-CNN model, the optimal parameters are selected from numerous experimental settings. Selection range of these parameters are determined by default values and certain characteristics of the real dataset.

#### 4.3.1 Parameters for learning network vector

The length *d* of vector $v_{i}^{\left(t\right)}$ appeared in Sect. 2.2, is set as 200, for the reason that the classification effect is not improved significantly with *d* increasing from 200 to 500. The top 20 nearest neighbors in $N_{v}^{\left(h\right)}$ of vertex *v* is selected to make up the matrix *T*_*v*_. Here, *h* equals 3.

#### 4.3.2 Common parameters of MHD-CNN and TRHD-CNN

Experimental results show that the two CNN models converge under 100 epochs, therefore we set the training termination condition to a fixed epoch value of 100. In our experiments, during each iteration, the learning rate is dynamically adjusted to accelerate the convergence, i.e. the learning rate is selected from the set {0.01, 0.05, 0.09, 0.13, 0.17, 0.21, 0.25} in descending order according to the training errors. In these two models, regularization term *L*_2_ and dropout rate are introduced to avoid over-fitting. These two parameters are assigned referring to the default values used in the relevant study [28], i.e. the correlation coefficient of *L*_2_ and dropout rate are set to 10e-4 and 0.5, respectively.

#### 4.3.3 Parameters of MHD-CNN

The maximum length of the directed edge weight vectors (i.e. *d*_*h*_) is derived from our transaction network based on real dataset. The maximum length value equals to 1359. To achieve optimal classification performance, the MHD-CNN model adopts the ReLU activation function in convolutional layers and max pooling function in pooling layers.

#### 4.3.4 Parameters of TRHD-CNN

In this part, we list the parameters of each component of TRHD-CNN in turn.

*A*. *Parameters of the convolutional component*: In the first-route of the TRHD-CNN model, the values of all parameters and the selected activation functions are consistent with those of the MHD-CNN model. In the second-route, the used parameter settings are listed in Table 3.

**Table 3:** Values of the second route parameters.

Parameter	used values
*c*_1_	4
*k*_1_	100
shapes of *n*_1_	{1 × *n*_1_, *n*_1_ = 2, 3, 4, 5}
*p*_1_	3
*c*_2_	3
*k*_2_	50
shapes of *c*_2_	{1 × *n*_2_, *n*_2_ = 2, 3, 4}

In its first convolutional layer, the number of convolution kernels *c*_1_ and the number *k*_1_ of each kind of kernel are set as 4 and 100, respectively. And then the shapes of *c*_1_ convolution kernels are set as {1 × *n*_1_, *n*_1_ = 2, 3, 4, 5}. The width of pooling window of the first pooling layer *p*_1_ is set as 3. Similarly, in the second convolutional layer, *c*_2_ and *k*_2_ are set to 3 and 50, respectively, and these *c*_2_ convolution kernels shaped like {1 × *n*_2_, *n*_2_ = 2, 3, 4}. The following pooling layer adopts a pooling window in shape of *m* × *n*, where *m* and *n* are the number of rows and columns of the corresponding input feature map, respectively.

*B*. *Parameters of the classification component*: The experimental results show that the concatenated vector, when *K*_1_ and *K*_2_ are set as 100 and 50 respectively, achieves the best results in classifying bank accounts.

### 4.4 Analysis of the classification performance

In this section, we analyze the classification performance of the proposed CNN models from two aspects, evaluating the impacts of different parameters, and comparing classification performance with other models.

#### 4.4.1 Evaluating different architecture parameters of TRHD-CNN

We test the influence of the value of parameters *K*_1_ and *K*_2_, and the number of convolutional layers on the classification effects of TRHD-CNN, respectively.

**Fig 8:**
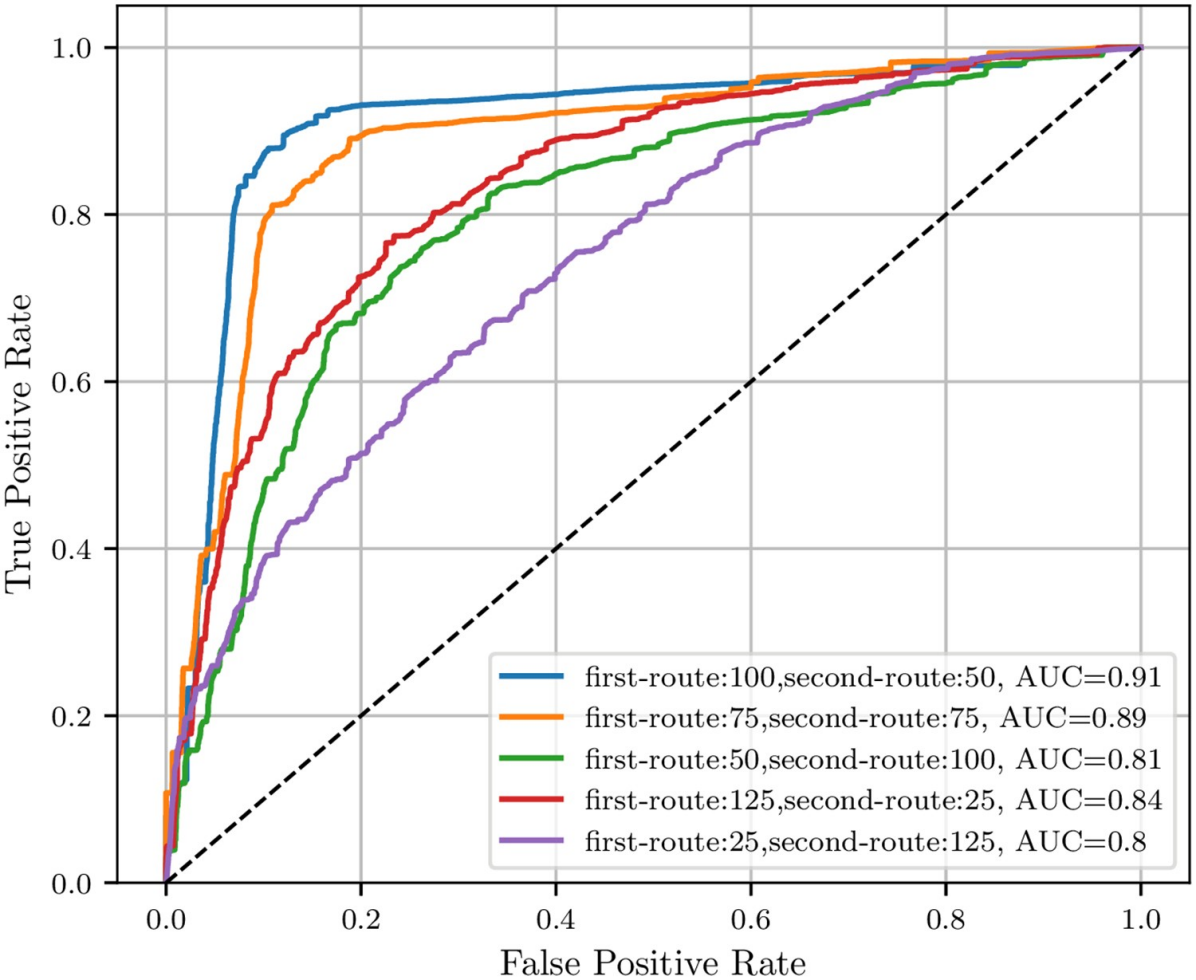
The ROC curves of TRHD-CNN with different output vector lengths of the two routes.

(1) Tuning the values of *K*_1_ and *K*_2_. In this section, we tune the parameters *K*_1_ and *K*_2_ to receive the best classification results for TRHD-CNN. The details are shown in Fig 8.

In the legend, the first-route means the parameter *K*_1_, and the second-route corresponds to *K*_2_. The value of AUC means the area under the curve. As well known, the greater the AUC value is, the better the classification performance of the model will be. As shown in Fig 8, TRHD-CNN model achieves the best results with *K*_1_ = 100 and *K*_2_ = 50. Therefore, we set *K*_1_ to 100 and *K*_2_ to 50 in TRHD-CNN. The result indicates that the impact of the typological network data is greater than that of the time series data on classification effects, which further illustrates the advantage of our improved two-route framework.

(2) Adding extra convolutional layers. It is found that the TRHD-CNN model receives the best results when the first-route contains one convolutional layer followed by a pooling layer, meanwhile the second-route contains two convolutional layers and each layer is connected to a pooling layer. The reason why no significant improvement in classification performance is made when increases the convolutional layers, may be that the features is already well described by TRHD-CNN model by shallow network structure.

#### 4.4.2 Comparing the classification performances of different models

In this section, three groups of experiments are carried out on the three models, the LOF method [29], the MHD-CNN model and the TRHD-CNN model, for testing their performance in classifying bank accounts.

Note that we test several traditional abnormal account detection methods based on statistical features in our prior research [29]. [29] focuses on detecting bank accounts by using traditional classifiers with novel devised three kinds of behavioral features. In [29], the classification problem on an imbalance dataset is seen as an abnormal detection problem. The utilized classification features, being extracted from their financial time series transaction records, are grouped into three categories: transaction statistical features, network behavioral features and periodic behavioral features. A comparative analysis of several classical classifiers, one-class SVM, isolation forest (IForest), local outlier factor (LOF) and robust covariance (RC), is made in [29]. Given a predefined outlier fraction, the experiment results show that IForest achieves the best F1-score, i.e. 58%. It is obviously that the traditional abnormal account detection methods are uncompetitive with the following ones.

Therefore, in the following paper, the performance of IForest method is regarded as a benchmark, and the other two models are tested with different parameter values as mentioned in *Sect*. *4*.*3*. In particular, for IForest method, three values of a necessary threshold parameter, i.e. 0.01, 0.08, 0.1, meaning the outlier factor are selected in our experiments. The optimum outlier factor, which achieves the best result on the training set is selected for IForest. The classification results of the three models on the dataset are shown in Table 4, where the optimal results of MHD-CNN and TRHD-CNN are exhibited.

**Table 4:** The classification results of the three models. The numbers in parenthesses refer to the corresponding average results on training set.

Models	No.	Fractor	Accuracy (%)	Precision(%)	Recall(%)	F1-score(%)
IForest	1	0.08	93.54 (93.81)	64.1 (63.16)	57.14 (67.86)	60.42 (65.43)
MHD-CNN	2	∖	96.85 (97.19)	84.91 (86.31)	77.14 (80.14)	80.84 (83.11)
TRHD-CNN	3	∖	97.39 (97.7)	86.75 (89.16)	82.29 (83.43)	84.46 (86.2)

As shown in Table 4, both CNN models achieve better results than the traditional statistically methods (i.e. IForest), proving the superiority of deep learning methods in mining classification features. The reason may be the statistical features are manual extracted, which are static during the classification process. However, the classification procedure of CNN model contains a feedback process, which modifies the adopted classification features dynamically. Moreover, the MBGD algorithm, a back-propagation method used for update the parameters, ensures the CNN models converges to the minimum loss state. Therefore, CNN always chooses the most effective features according to specific classification tasks, and learning the optimal classification parameters, automatically. The improvement of CNN is not intuitively obvious, referring to the generally high accuracies of the three methods. It should be noted that the recall is a more important evaluating indicator in a fraud identification scenario. Moreover, the high accuracy is caused by the imbalance between the number of illegal samples and normal samples. Table 5 gives details of the methods’ classification results on test set, where the indicators true positive, false negative, false positive, true negative are respectively denoted as TP, FP, FN, TN. Each time, one fifth dataset is randomly selected as test set, containing 175 MLM accounts and 1854 normal accounts. As presented in Table 5, 144 MLM accounts are identified by TRHD-CNN, in contrast with the amount 100 IForest recognized. And yet, TRHD-CNN makes significant improvement over MHD-CNN in TP.

**Table 5:** The confusion matrix of the three methods.

Models	No.	TP	FP	FN	TN
IForest	1	100	56	75	1798
MHD-CNN	2	135	24	40	1830
TRHD-CNN	3	144	22	31	1832

On the whole, the TRHD-CNN is higher than the MHD-CNN in precision, recall and F1-score, indicating different types of convolution kernels have advantage of extracting more useful features.

The results in Table 4 show that TRHD-CNN better than the other methods on accuracy and recall obviously. To further investigate the superiority of TRHD-CNN, we perform the *Paired Samples T Test (SPSS)* for statistical significance. Four times five-fold validation was performed on the data, and the accuracy results on the testing set are used for SPSS. Here, IForest and MHD-CNN are compared with TRHD-CNN respectively. The results are shown in Table 6. According the definition of Probability Value (*p-value*), the smaller the p-value, the more significant the difference is. Moreover, we can draw a conclusion that the difference is statistically highly significant when *p-value* is smaller than 0.01. Based on the *p-values* in Table 6, we reject the null hypothesis, indicating a definite improvement provided by TRHD-CNN.

**Table 6:** Results of SPSS on accuracy.

Classifier Pairs	No.	Mean difference $\bar{d}$	Standard Deviation *s*_*D*_ of $\bar{d}$	P-value
IForest vs TRHD-CNN	1	0.0403	7.18E-05	1.33E-21
MHD-CNN vs TRHD-CNN	2	0.0054	2.89E-06	9.27E-16

**Fig 9:**
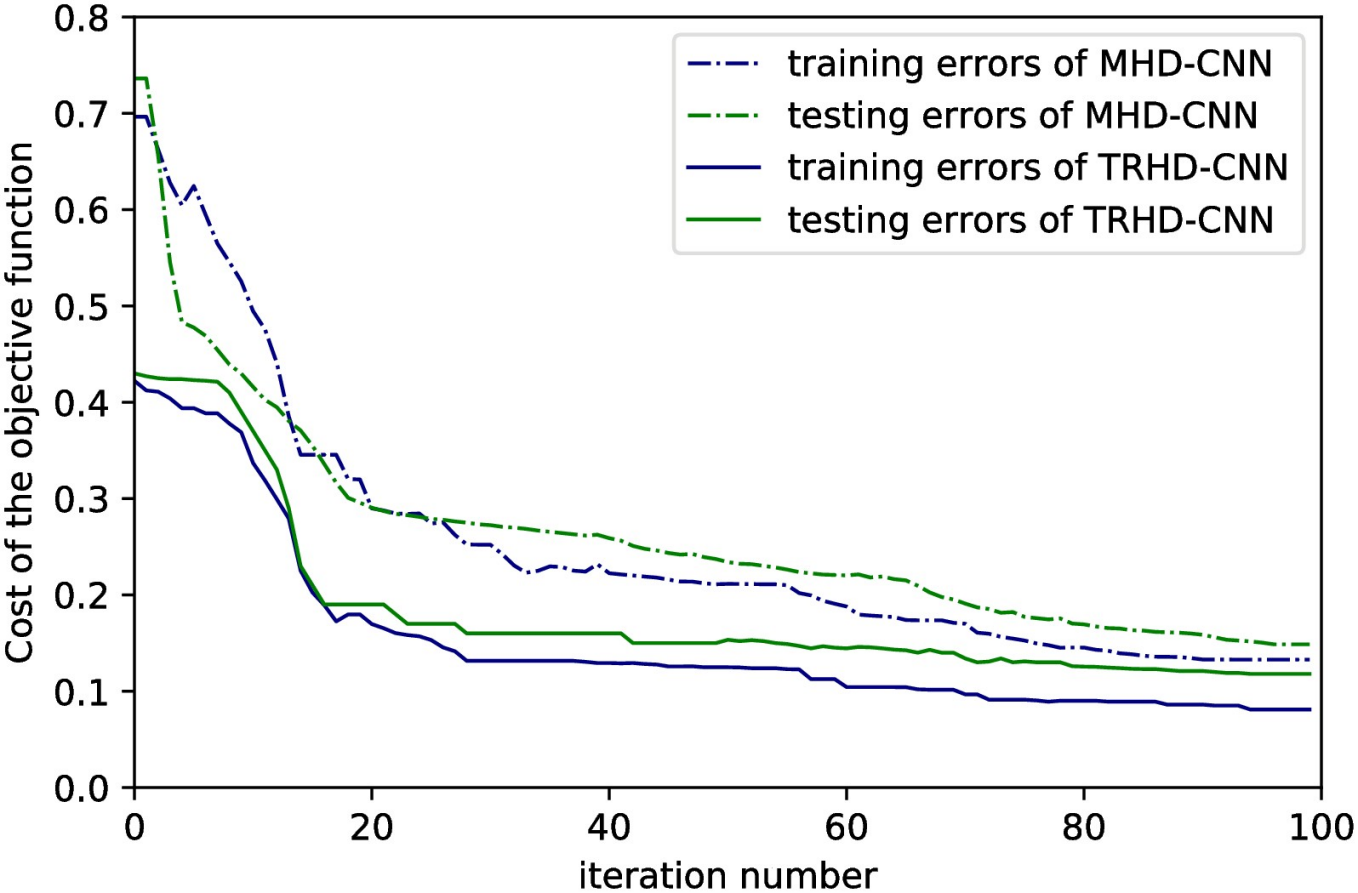
Training and testing errors of TRHD-CNN and MHD-CNN with various iteration times.

In the process of testing the proposed CNN models, we adopt the method of dynamically selecting learning steps to accelerate the convergence of the learning model. Fig 9 shows the changes of training and testing errors with increasing iteration times for CNN models.

The x-axis and the y-axis represent the number of iteration times and the cost values of the objective function, respectively. As shown in Fig 9, along with the iteration number increases, all curves diminish sharply at the beginning, then decline in decreased speeds, and finally converge to stable values. Obviously, the training errors and testing errors of the TRHD-CNN model are lower than those of the MHD-CNN model in general, and the reason is that the TRHD-CNN model is helpful in extracting more useful classification features. In addition, using the strategy of dynamic selection of step size, the fluctuations of the cost curves almost disappear after a certain number of iterations (beginning at the 80th iteration). Hence, reducing the step size after a specific iteration number is helpful for the objective function to converge to an optimal value correctly and rapidly.

*In summary*, the experiment results indicate that the existing methods cannot take advantage of the information, embedded in the time series transaction data, efficiently. While, the TRHD-CNN model, which employs a two-route convolution structure for extracting the specific classification features, gains a significant advantage over other algorithms. Its superiority in the recall indicator proves the complementary of local topological structure features and time series transaction features in describing the category of an account. And yet, to say that TRHD-CNN is time consuming in learning a new coming account’s classification features.

## 5 Conclusion

This paper considers the CBA problem by using features come from two kinds of heterogenous data separately. It is found that the two data are complementary in describing the classification category of an account. This paper develops a two-route TRHD-CNN model, which adopts a devide and conquer strategy to train the two kinds of input matrices simultaneously, and employs a two-route convolution structure for extracting the specific classification features. During its training, TRHD-CNN utilizes local topological features to describe the relationship information and time series transaction features to extract the spending information. This paper develops a DirectedWalk algorithm to learn the network vector of vertices in a directed and dynamic network. The experimental results on a real dataset show that TRHD-CNN has a significant advantage over other algorithms.

## 6 Future work

In our research, we have found that sub-sequence of the time series transaction records is of great significance for identifying the category of an account. Therefore, in our future research, we intend to use phase space theory to extract the sub-sequence features to improve the classification effects. In addition, the Long Short-term Memory (LSTM) model has been proved very suitable for classifying data with their time series characteristics. Thus, how to combine LSTM to process time series transaction data to improve the TRHD-CNN model is another problem we will solve in the next step.

Currently, the real bank account dataset is huge in volume but rare in labelled samples, therefore, we aim to adopt semi-supervised strategy to improve the TRHD-CNN model.

In summary, the research on account classification using transaction data is still in its infancy. It’s our continuous goal to explore high efficiency classification models with the transaction data.
